# Attitudes toward Pursuing Genetic Testing among Parents of Children with Autism Spectrum Disorder in Taiwan: A Qualitative Investigation

**DOI:** 10.3390/ijerph19010118

**Published:** 2021-12-23

**Authors:** Zihan Zhang, Justin Kramer, Haocen Wang, Wei-Ju Chen, Tse-Yang Huang, Yann-Jang Chen, Tung-Sung Tseng, Lei-Shih Chen

**Affiliations:** 1 Department of Health and Kinesiology, Texas A&M University, College Station, TX 77843, USA; zihan2017@tamu.edu (Z.Z.); justin.kramer@tamu.edu (J.K.); hwang472@tamu.edu (H.W.); 2 Department of Psychology, The University of Texas Permian Basin, Odessa, TX 79762, USA; chen_w@utpb.edu; 3 Department of Special Education, National Tsing Hua University, Hsinchu 30013, Taiwan; tyhuang@mail.nd.nthu.edu.tw; 4 Department of Life Sciences and Institute of Genome Sciences, National Yang Ming University, Taipei 11211, Taiwan; yjchen@ym.edu.tw; 5 Behavioral and Community Health Sciences Program, School of Public Health, Louisiana State University Health Sciences Center, New Orleans, LA 70112, USA; ttseng@lsuhsc.edu

**Keywords:** autism spectrum disorder, parents, perceptions, knowledge

## Abstract

Background: The diagnosis of autism spectrum disorder (ASD) cases is increasing in Taiwan. Genetic testing for children with ASD offers several potential benefits and is available with out-of-pocket expenses. Parents play a pivotal role in having their children with ASD tested; therefore, understanding their perceptions of, and perceived barriers to genetic testing is vital. Methods: Semi-structured interviews were conducted with 39 parents of children with ASD in Taiwan. Interviews were recorded and transcribed verbatim. NVivo 12 software (QSR International, Doncaster, Australia) was used to facilitate an inductive coding methodology. Results: The majority of participants (74.4%) supported ASD genetic testing for their children with ASD, citing reasons such as clarifying ASD etiology, well-informed family planning, contributing to ASD research, and early ASD detection and intervention. Others indicated that they were either against such testing (17.9%), or unsure (7.7%) about whether to take their children with ASD for genetic testing. Those who were opposed reported that their main concerns related to perceptions of no value of genetic testing, potential for family conflict, and financial difficulties. Conclusions: Most of the parents of children with ASD that we interviewed expressed favorable views of ASD genetic testing. There exists a need to increase parental access to education and counseling, and to include testing coverage in Taiwanese national health insurance.

## 1. Introduction

Autism spectrum disorder (ASD), characterized by symptoms that generally present before the age of 2, is a developmental disorder characterized by communication, behavioral and social interaction challenges, restricted interests and repetitious behaviors, and difficulty functioning in traditional work or school environments [1,2]. In Taiwan, which has a population of approximately 23.4 million [3], the prevalence of ASD has nearly tripled in the past 14 years, increasing from 5359 in 2005 to 15,439 cases in 2019 [4]. This increase in ASD diagnoses has been linked to many factors, including better ASD awareness, changes in ASD diagnostic criteria, and improved access to ASD-specific services [5,6,7].

Research has shown that ASD is highly associated with genetic causes [2,8], with heritability ranging between 37% and 50% [9,10]. As such, genetic testing plays an important role for both the children with ASD and their families. The main benefits of genetic testing for children with ASD include the potential of clarification of ASD etiology, provision of personalized medical treatments and management strategies, and the identification of comorbidities and associated complications [11,12,13]. As parents of children with ASD are at increased risk of having additional children with ASD in future pregnancies [10,14], ASD genetic testing results may also assist in making informed family planning decisions [13].

In Taiwan, genetic testing (chromosomal microarray (CMA) and whole exome sequencing) is available for children with ASD with out-of-pocket expenses ranging between 18,000 NTD (~600 USD) and 30,000 NTD (~1000 USD) [15,16]. Pediatric neurologists, psychiatrists, and geneticists are the main healthcare providers who offer ASD genetic testing to parents of children with ASD. However, while parental consent is required to have children undergo ASD genetic testing, researchers have yet to explore Taiwanese parents’ views regarding pursuing genetic testing for their children with ASD. Studies based in other nations including the United States (U.S.) [17,18], Norway [19], Israel [20], and Turkey [21], have explored parental perceptions of genetic testing for their children with ASD. Research has found that the majority of parents are in favor of ASD genetic testing, often citing reasons such as the utility of testing results to help explain the etiology of their child’s ASD, improve their child’s ASD-related care, advance ASD research and treatment, and make better informed future family planning decisions (for both the parents and for future generations) [17,18,19,20,21,22].

Noting the rapidly increasing rate at which ASD is being diagnosed in Taiwan [4], there exists an urgent need to understand the perspectives of parents of children with ASD regarding genetic testing. Therefore the aim of this effort, which is to the best of our knowledge a first-of-its-kind study, is to explore parental willingness to pursue genetic testing for their children with ASD in Taiwan. This study not only offers unique insight into the views of Taiwanese parents of children with ASD, but it also allows for transnational and transcultural comparisons to be made between groups of parents across the world.

## 2. Materials and Methods

### 2.1. Study Design

A qualitative approach utilizing conventional content analysis methods was employed. We used a qualitative approach because little was known about Taiwanese parents’ viewpoints regarding pursuing genetic testing for their children with ASD. Specifically, we conducted in-person, face-to-face interviews to collect in-depth qualitative data on parental attitudes regarding pursuing ASD genetic testing for children diagnosed with ASD. This present study is part of a larger qualitative research project examining the perceptions of the etiology of ASD, ASD genetic testing, and the educational needs of Taiwanese parents of children with ASD [16,23,24]. The Institutional Review Board at Texas A&M University approved the study protocol prior to any data acquisition. 

As researchers are the “instrument” of qualitative research [25], it is important to present the values, training, and background of our research team. Our research team members have been working as trained professionals in genetics/genomics, public health, and special education for many years. We have significant experience studying underserved populations and conducting social and behavioral science research related to ASD. Most of our research team members are bilingual (English and Mandarin) and understand Taiwanese and Asian culture well. One of the research team members is also a Taiwanese mother of a child with ASD. This unique background helps better inform our research team and provides us with an insightful perspective towards the topics explored in this study.

### 2.2. Participants and Recruitment

Taiwanese parents who have at least one child diagnosed with ASD were the target population of the present study. Prospective participants were recruited with the assistance of the Taiwan Autism Society, the Hsinchu Association of Autism, the Changhua County Autism Association, and the Taichung County Autism Association. Snowball sampling methods were then used to recruit additional participants. Informed consent was obtained from all participants in this study.

### 2.3. Data Collection Procedure

Our semi-structured interviews were facilitated using an interview guide that was developed based on prior research [26,27] and reviewed by experts in the fields of qualitative research, genetics, special education, and health promotion. All interviews were conducted and audio recorded in a quiet and private place; field notes were also taken during interviews. Data collection continued until thematic saturation was achieved. A total of 39 parents of children with ASD were interviewed, with each interview averaging 131 min (SD = 75.6 min). Additionally, socio-demographic information was collected from participants and their children with ASD using a short paper-and-pencil survey. Each parent was offered an incentive of 500 NTD (~16.7 USD) for participating in the study.

### 2.4. Data Analysis

Upon the completion of data collection, all interviews were transcribed verbatim for data analysis. A conventional content analysis approach and inductive coding strategy was used. Content analysis allows researchers to both analyze the data qualitatively and quantify the presence of themes within the qualitative data. This approach aided us in (1) identifying participants’ reasons for pursuing or not pursuing ASD genetic testing for their children with ASD and (2) counting the frequencies of the identified reasons [28]. NVivo 12.0 software (QSR International, Doncaster, Australia) was used to facilitate the analysis. All codes and themes were derived directly from our collected data. In particular, two authors (Z.Z. and H.W.) independently read the transcripts several times to obtain a general idea of the participant responses and extract codes from the data. The emerging codes were then combined into themes based upon similarities and differences. All codes and themes were discussed for verification and agreement between these two authors. The refined codes and themes were further checked and verified by another author (L.C.), who was also the interviewer for this study.

**Figure 1:**
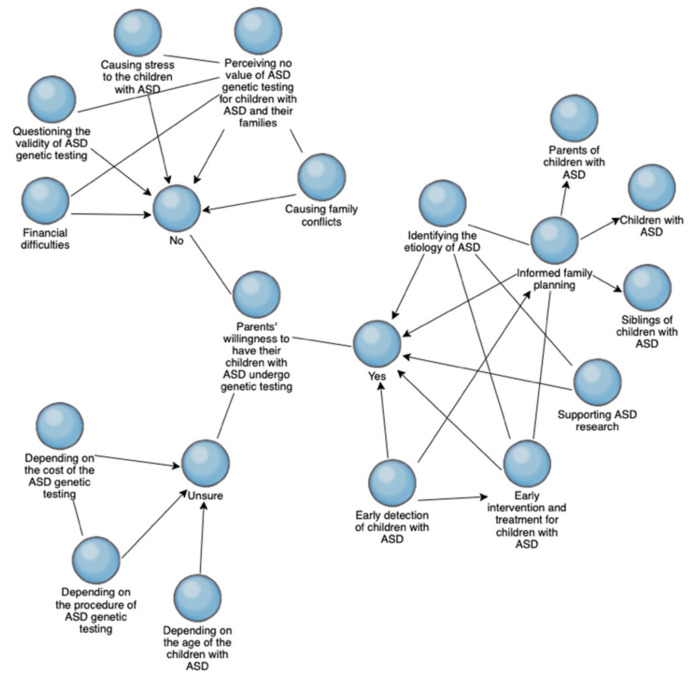
Conceptual map of parents’ willingness to have their children with ASD undergo genetic testing.

In addition, we created a concept map (Figure 1) using NVivo 12.0 to illustrate the coding results, provide a visual presentation of all the themes generated from our data analysis, and present differences across groups of participants [29]. The concepts (blue circles) in Figure 1 represented the research focus and the themes identified through data analysis. The relationships among these concepts were indicated by lines and arrows. Participants were divided into three groups based on their perspectives about pursuing genetic testing for their children with ASD.

## 3. Parental Attitudes toward Pursuing ASD Genetic Testing for Their Children with ASD

To explore the parents’ attitudes regarding ASD genetic testing, we first asked how much they knew about ASD genetic testing. Slightly more than half of the parents (53.8%) said they had previously heard about ASD genetic testing prior to this study. Eighteen parents (46.2%), however, were not familiar with this testing. Participants were then asked if they would be willing to have their children undergo ASD genetic testing. Parents’ attitudes toward pursuing ASD genetic testing for their children with ASD were mixed, but most (74.4%) expressed a favorable view. However, several parents (17.9%) opposed pursuing ASD genetic testing, and a few parents (7.7%) were unsure about whether or not to let their children with ASD undergo this testing. The details of the emerged themes are described as follows:

### 3.1. Intent to Pursue ASD Genetic Testing

The majority of participants (74.4%) stated that they would want their children with ASD to undergo genetic testing. Parents’ supporting reasons for pursuing ASD genetic testing are listed in Table 1 and are described in order of frequency.

*Identifying the etiology of ASD (41.4%).* Among the participants who had favorable attitudes toward ASD genetic testing, the most frequently identified reason was to discover the etiology of ASD through genetic testing. Particularly, parents wanted to know if their children’s disease was inherited from them. For example, a mother, whose 11-year-old son was diagnosed with ASD at the age of 6, told us that she would like her son to undergo ASD genetic testing because her husband’s family had a history of marriages between close relatives. She had always suspected this had caused her son’s ASD by explaining that:

“I’m always thinking… it [ASD] may be inherited…His [her son’s] father’s side has some problems…They [her husband’s relatives] haven’t banned marriages between close relations…their marriage was arranged by the family.” (Participant #3, female; income: less than 600 K NTD (<20 K USD); college and above degree)

Similarly, other participants were interested in ASD genetic testing because they wanted to find out whether the cause of their child’s ASD was nature (inheritance) or nurture. For example, one father who was a schoolteacher told us that his wife’s delivery had been delayed due to a doctor’s error, and that his wife thought the delay in delivery might have contributed to their child’s ASD. Yet, he had also learned from his colleagues, who were special education teachers, that genetic factors could play a role in causing ASD. As a result, he would like to have his child with ASD undergo genetic testing to understand the etiology of the condition. According to this father:

“My wife is always guessing…she often says the birth of our son with ASD was delayed…She thinks that’s the reason causing ASD since we can’t find any other causes…From observing special education classes and discussing with my special education teacher colleagues, I feel like genetic factors may also have contributed to our son’s ASD.” (Participant #39, male; income: more than 1 M NTD (>33 K USD); college and above degree)

*Informed family planning (37.9%).* The second most frequently mentioned reason for undergoing genetic testing pertained to family planning, with parents noting that ASD genetic testing could help them and their children (both those with ASD and those without) make informed family planning decisions. For example, one participant reported that he hoped ASD genetic testing results could help him to prevent having another child with ASD, stating that:

“I want to know if ASD is hereditary in case the same thing [having a child with ASD] happens again in the future.” (Participant #8, male; income: less than 600 K NTD (<20 K USD); college and above degree)

Furthermore, parents in our sample overall wished that their children with ASD could eventually get married and have their own children. Nevertheless, they were also worried that their children’s ASD might be passed on to the next generation. A father, for instance, explained that if genetic testing showed that his daughter’s ASD was inherited, he would use this information when making future family plans for his daughter. He was concerned that his daughter might also have a child with ASD, emphasizing that taking care of a child with ASD would be a big responsibility and challenge for her.

Concerns regarding having offspring with ASD also made several parents think that ASD genetic testing could help inform the future family planning decisions for the siblings of the children diagnosed with ASD. A mother, for example, stated that if ASD genetic testing could show which specific gene was problematic, she could tell her other healthy children so that they could be mentally prepared when they are planning to have children in the future. According to this mother:

“If I know which gene has [a] problem, I will tell my first and third kids who are healthy about this…so in the future, when they want to have children, they can prepare for this.” (Participant #17, female; income: between 600 K and 1 M NTD (20–33 K USD); college and above degree)

*Supporting ASD research (24.1%)*. Interestingly, some parents noted that they would pursue ASD genetic testing to support ASD research. This is illustrated by the comment of one interviewee who explained that:

“If researchers let me know why they want to perform genetic testing for my child with ASD, I would have my child undergo it…I would provide the sample to assist research.” (Participant #5, male; income: more than 1 M NTD (>33 K USD); college and above degree)

The desire to help research was echoed by several participants. Although these participants did not believe that genetic testing was necessary for their children with ASD and doing this might also cause family conflicts, they would still have their children tested. This is because they believed that the test findings could contribute to ASD research and help other children with ASD. According to one mother:

“I would let my child do it [genetic testing] if it can help more children with ASD…I can provide a sample for the research…but I don’t need to know the results… suppose it turns out to be me or my husband who causes my son’s ASD, should we blame each other?” (Participant #4, female; income: between 600 K and 1 M NTD (20–33 K USD); college and above degree)

*Early intervention and treatment for children with ASD (10.3%)*. Several participants reported that they would like their children with ASD to undergo genetic testing for the purpose of early intervention and treatment. For example, a mother of a son with mild ASD mentioned that all parents who have children with ASD should have their children undergo genetic testing. By doing this, their children could receive early intervention and treatment, which would subsequently lead to better physical and cognitive development.

*Early detection of children with ASD (6.9%).* A few parents reported that early detection was the reason they were in favor of pursuing ASD genetic testing. For instance, a mother of a daughter diagnosed with ASD mentioned that ASD genetic testing might help with the early detection of ASD, which would further benefit her child. She complained that some people, typically men like her husband, did not believe their children had ASD. Rather, they felt that these children would eventually become normal as they grow up. In her view, genetic testing might help to provide a clear diagnosis and detection of ASD as early as possible. 

### 3.2. Unwillingness of Pursuing ASD Genetic Testing

Table 2 summarizes some (17.9%) parents’ reasons for opposing taking their children for ASD genetic testing. The themes that emerged are described in detail below:

*Perceiving no value of ASD genetic testing for children with ASD and their families (85.7%)*. Among the participants that were against ASD genetic testing, most, regardless of the current age of their children with ASD, felt that this testing was unnecessary. They believed that the testing results would not improve the affected child’s current conditions and future outcomes. An illustration of this can be seen in the below excerpt of a conversation between the interviewer and one mother whose son was diagnosed with ASD and currently aged two:

“Interviewer: Would you be willing to take your child with ASD for genetic testing?

Mother: Not necessary. I don’t feel the need now. Because I don’t know…I mean so what after undergoing genetic testing.

Interviewer: Are you saying that there is nothing you could do?

Mother: Yes. So, there is no need for my child to undergo genetic testing.”

*Causing family conflicts (28.6%)*. Some parents were worried that the ASD genetic testing results might create family conflicts, so they did not want their children with ASD to undergo genetic testing. In particular, one interviewee was afraid that if the genetic testing results found that the child’s ASD was inherited from one of the parents, it was very likely to lead to conflict and divorce. The parent who had the ASD genes might be blamed by the partner for causing their child’s ASD. 

*Causing stress to the children with ASD (14.3%).* Concerning that the genetic testing procedure might cause stress to the children with ASD was another refusal reason for ASD genetic testing. As the genetic testing process might involve drawing blood, this could potentially be very difficult to do. One mother explained that her son was sometimes not very cooperative when it came to blood draws, so the process might cause him to become injured. 

*Financial difficulties (14.3%)*. One of the parents’ concerns regarding having children with ASD undergo genetic testing was related to its cost. One mother particularly mentioned that having a child with ASD requires someone to be at home at all times. Since having a child with ASD, she had not been able to find a full-time job, and her current job and salary were not stable. As such, she could not afford the cost of ASD genetic testing.

*Questioning the validity of ASD genetic testing (14.3%).* The final refusal reason was parents’ concerns regarding the technique and accuracy of ASD genetic testing. One father, who held a graduate degree, reported that he did not know whether ASD genetic testing was a valid and reliable technology. He also questioned the accuracy of the testing results, specifically their potential for producing incorrect findings.

### 3.3. Unsure about Whether to Pursue ASD Genetic Testing

A few parents (7.7%) were unsure about whether or not to take their children with ASD undergo genetic testing. We listed the emergent themes in Table 3, from the highest frequency to the lowest, below:

*Depending on the procedure of ASD genetic testing (66.7%)*. Among the parents who were undecided if they would like to pursue ASD genetic testing for their children with ASD, most reported that it would depend on the testing procedure. Specifically, one interviewee reported that he would only allow his son with ASD to have his blood drawn once by explaining that:

“It’s fine to draw blood once, but I would hesitate to take my son for genetic testing if it requires to draw blood several times.” (Participant #15, male; income: more than 1 M NTD (>33 K USD); college and above degree)

*Depending on the cost of ASD genetic testing (33.3%)*. The cost of ASD genetic testing was an important concern. One father remarked that the decision of whether to pursue ASD genetic testing or not would depend on cost. If it was only a few thousand NTD, his son could be tested. However, if it cost more than ten thousand NTD out-of-pocket, it was impossible to go through with the test.

*Depending on the age of the children with ASD (33.3%).* The age of the children with ASD was the other factor affecting parents’ decision in taking their children for testing. One mother, who had a 9-year-old son with mild ASD, reported that the age of the child with ASD would be an important testing decision-making factor. She would pursue ASD genetic testing for her son because he was old enough. Yet, she believed that ASD genetic testing would be unnecessary for her son and other children with ASD when they were very young, because they might get better as they grew up.

## 4. Discussion

This study, which to the best of our knowledge is the first of its kind, explores Taiwanese parents’ intention to pursue genetic testing for their children with ASD by conducting in-depth, face-to-face interviews with 39 parents. Nearly three-quarters of the Taiwanese parents of children with ASD whom we interviewed held favorable views towards the use of genetic testing for their children. This finding is consistent with previous research carried out in Turkey [21], Norway [19,22], Israel [20], and the U.S. [17,18]. Specifically, the majority of the factors that we found to be associated with positive perceptions of genetic testing have also been reported by parents of children with ASD in other studies, including (1) better understanding of the causes of their child’s ASD [17,20,21,22], (2) increasing their ability to make informed family planning decisions in the future [17,18,20], (3) contributing to ASD research [17,20,21,22], and (4) detecting ASD early and initiating interventions [17,19,21].

Of note, some participants in our study explicitly mentioned early detection and intervention as being motivating factors that could impact their decision to have their child undergo ASD genetic testing. Nevertheless, this reasoning conveys a lack of understanding about the purpose and scope of ASD genetic testing. Findings consistent with our results were also noted in the U.S., Norway, and Turkey [17,19,21]. In fact, as children who undergo ASD genetic testing are diagnosed with ASD prior to the initiation of testing, the genetic tests themselves do not diagnose ASD. Rather, the primary purpose of testing is to identify the genetic causes and potential comorbidities associated with the children’s previously diagnosed ASD, which can then be leveraged to make more informed decisions and tailor care to the specific needs of the children [11,12,13]. Accordingly, our results highlight the need for increased genetic education and counseling for parents of children with ASD in Taiwan.

While most of the Taiwanese parents we interviewed were in favor of ASD genetic testing for their children, about one-fifth reported that they were opposed to it. The most common reason for this opposition was a perception that the results of genetic testing had limited utility. This finding has also been noted in other studies conducted in the U.S. [17,30], Israel [20], and Norway [19]. For example, Hendel et al. [20] found that although ASD genetic testing is fully covered by Israel’s National Insurance Law, 55% of parents of children with ASD cited the test’s “irrelevance” as being their reason for refusing the test [20]. Although ASD genetic testing results may be negative or clinically inconsequential, positive genetic testing findings can have a substantial impact for children and families, as they can shape a clinical care approach, provide an explanation of ASD etiology, and inform parents of their risk of having another child with ASD [31]. For example, Reiff et al. [32] found that parents who had their children undergo CMA reported that it was beneficial to be informed of the findings resulting from CMA, irrespective of whether or not the results had clinical utility.

The possibility of genetic testing creating family conflict was another major theme that should be explored in future research, as not only was this reasoning raised in our sample of Taiwanese parents of children with ASD, but it was also cited among parents in the U.S. [17] and Norway [19]. Those in our sample who expressed concern, grounded their thoughts in the belief that genetic testing might identify one of the parents as being responsible for the child’s ASD condition, which could then lead to strife and marital discord. Accordingly, pre- and post-test genetic counseling and education with marriage support and counseling should be provided to parents to minimize the potential of assigning blame to one parent. Moreover, families with special needs often experience discrimination or inequity in Asian culture [33], it is important to promote cultural changes and create a friendly and supportive environment for families with special needs.

Though not as frequently reported, participants also explained that the associated medical costs would factor into their decision to pursue ASD genetic testing. One mother reported that the cost of ASD genetic testing would prevent her from pursuing genetic testing for her child with ASD. Understandably, financial hardship has been cited in several studies as a barrier to ASD genetic testing [18,19,30], and highlights the need for parents of children with ASD to receive increased financial support and education. In Taiwan, national health insurance offers universal, mandatory coverage to 99.8% of the Taiwanese population [34] but it does not cover the cost of ASD genetic testing. Given that the average annual income per person in Taiwan is about $24,557 USD per year (~$2,046 USD per month) [35], and the cost of ASD genetic testing is between 18,000 NTD (~600 USD) and 30,000 NTD (~1,000 USD) [15,16], this significant expense potentially represents a major burden for families affected by ASD. With the increasing number of children that are being diagnosed with ASD in Taiwan, it is recommended that the Taiwanese national health insurance either fully or partially covers the expense of ASD genetic testing, a change that could potentially increase genetic testing uptake.

It is important to note that despite the availability of ASD genetic testing in Taiwan, nearly 50% of the parents of children with ASD in our sample were unaware of the availability of genetic testing for their children. Such findings have also been observed in other studies [17,18,36,37]. For example, Chen et al., [17] interviewed 42 Texas parents of children with ASD, and found that 63% of those parents had never heard of ASD genetic testing. Healthcare providers, especially pediatric neurologists, psychiatrists, and geneticists, should clearly communicate the availability of ASD genetic testing to their patients. ASD-related organizations in Taiwan, such as the Autism Foundation of the Republic of China and Taipei Parents Association of Autism, would be well-served to disseminate genetic testing information to their members, which might allow families affected by ASD to become more familiar with ASD genetic testing.

This study has two primary limitations, both of which are common to qualitative research and center on the potential generalizability of our findings. First, while our qualitative research frame is conducive to providing a much more thorough and in-depth analysis of parents’ motivations for engaging their children in genetic testing, it lacks the large sample size and statistical power that quantitative studies offer. To this end, our findings might not be representative of the views of all Taiwanese parents of children with ASD. Second, although we intended to recruit a diverse sample, most participants had one child with ASD, hold different marital statuses, and had a child with mild or moderate ASD. The perspectives of parents with multiple children with ASD, who are not married, and those who have children with severe ASD should be examined in future studies.

## 5. Conclusions

Genetic testing for children with ASD offers potential health benefits, such as the identification of comorbidities and the ability to tailor health management plans to the specific needs of affected children. Parents play a critical role in having their children undergo ASD genetic testing, as their consent is required to initiate the procedure. Understanding Taiwanese parental awareness and perceptions about genetic testing is crucial. Consistent with past research [17,18,36,37], this first-of-its-kind study (to the best of our knowledge) found that nearly half of the participants were unaware that genetic testing was an option for their child with ASD, which highlights an area that both healthcare agencies and ASD advocacy groups in Taiwan should seek to remedy. With approximately three-quarters of the participants responding favorably to the idea of ASD genetic testing for their children with ASD, making parents of children with ASD more aware of the genetic testing options available to them is very important. Moreover, some parents expressed concerns associated with ASD genetic testing, such as the perception of no value of ASD genetic testing results, the potential for family conflicts, and the financial burden of undergoing testing. These concerns should be addressed through both patient education and counseling, and in adjustments to Taiwanese national health insurance that allow for coverage of ASD genetic testing.

## Figures and Tables

**Table 1:** Reasons for pursuing ASD genetic testing among parents of children with ASD.

Reasons	Illustrative Quotation
Identifying the etiology of ASD (41.4%)	“I think this is the only reason. What is the exact cause of my son’s ASD? Because I don’t think I have encountered anything special during my pregnancy.” (Participant #26, female; income: between 600 K and 1 M NTD (~20–33 K USD); high school or less)
Informed family planning (37.9%)	“It is better to know if it’s the mother’s or father’s problem that causes our child’s ASD … there is absolutely no way for me to have another child with ASD…I don’t have enough time and money.” (Participant #8, male; income: less than 600 K NTD (<20 K USD); college and above degree)
Supporting ASD research (24.1%)	“If we can find a cure for ASD through genetic testing, it will benefit the future of other children with ASD.” (Participant #9, male; income: more than 1 M NTD (>33 K USD); college and above degree)
Early intervention and treatment for children with ASD (10.3%)	“After undergoing genetic testing… If children with ASD can receive early intervention and treatment, they can actually learn some skills…If you find out too late, it can probably lead to some serious problems.” (Participant #31, female; income: between 600 K and 1 M NTD (20–33 K USD); college and above degree)
Early detection of children with ASD (6.9%)	“It’s good to let the child undergo genetic testing after birth because autism can be detected early and reduce lots of time to diagnosis ASD.” (Participant #38, female; income: more than 1 M NTD (>33 K USD); high school or less)

ASD, autism spectrum disorder. Note: the sum of the themes is greater than 100% as some participants reported more than one reason to support ASD genetic testing.

**Table 2:** Reasons for opposing ASD genetic testing among parents of children with ASD.

Reasons	Illustrative Quotation
Perceiving no value of ASD genetic testing for children with ASD and their families (85.7%)	“I don’t think my child needs ASD genetic testing…I don’t know what I can do with it.” (Participant #35, female; income: between 600 K and 1 M NTD (20–33 K USD); college and above degree)
Causing family conflicts (28.6%)	“If the testing result turns out to be a genetic cause and it’s my problem, I’ll be discriminated against at home.” (Participant #14, female; income: less than 600 K NTD (<20 K USD); college and above degree)
Causing stress to the children with ASD (14.3%)	“I’m afraid the test will make my son feel uncomfortable.” (Participant #36, female; income: less than 600 K NTD (<20 K USD); high school or less)
Financial difficulties (14.3%)	“Why would I do that [taking my child for genetic testing]? My job and salary are not stable, and I can’t afford the test.” (Participant #32, female; income: less than 600 K NTD (<20 K USD); high school or less)
Questioning the validity of ASD genetic testing (14.3%)	“I’m not sure the technology [of ASD genetic testing] is ready and the results are accurate.” (Participant #24, male; income: more than 1 M NTD (>33 K USD); college and above degree)

ASD, autism spectrum disorder. Note: the sum of the themes is greater than 100% as some participants reported more than one reason to oppose ASD genetic testing.

**Table 3:** Reasons for being unsure about whether to pursue ASD genetic testing among parents of children with ASD.

Reasons	Illustrative Quotation
Depending on the procedure of ASD genetic testing (66.7%)	“I won’t allow my child to undergo the test if it’s invasive…but it’s fine if it just draws the blood.” (Participant #28, female; Income: between 600 K and 1 M NTD (20–33 K USD); college and above degree)
Depending on the cost of the ASD genetic testing (33.3%)	“It’s fine for a few thousand NTD… but there is no way for my son to undergo ASD genetic testing if it cost more than ten thousand NTD” (Participant #15, male; Income: more than 1 M NTD (>33 K USD college and above degree)
Depending on the age of the children with ASD (33.3%)	“If my child was very young, I probably won’t take him to be tested.” (Participant #16, female; Income: between 600 K and 1 M NTD (20 K to 33 K USD); college and above degree)

ASD, autism spectrum disorder. Note: the sum of the themes is greater than 100% as some participants reported more than one reason.

## Data Availability

The data are not publicly available as data sharing was not included within the original study ethics submission or participant consent form.
